# Interruption of Capsular Polysaccharide Biosynthesis Gene *wbaZ* by Insertion Sequence IS*903B* Mediates Resistance to a Lytic Phage against ST11 K64 Carbapenem-Resistant Klebsiella pneumoniae

**DOI:** 10.1128/msphere.00518-22

**Published:** 2022-11-15

**Authors:** Xin Yin, Qingqing Fang, Zhiyong Zong

**Affiliations:** a Center of Infectious Diseases, West China Hospital, Sichuan University, Chengdu, People’s Republic of China; b Center for Pathogen Research, West China Hospital, Sichuan University, Chengdu, People’s Republic of China; c Division of Infectious Diseases, State Key Laboratory of Biotherapy, Chengdu, People’s Republic of China; d Medical Center of General Practice, West China Hospital, Sichuan University, Chengdu, People’s Republic of China; University of Nebraska Medical Center

**Keywords:** phage therapy, *Klebsiella pneumoniae*, K64, capsule polysaccharide, phage resistance, *Klebsiella*, bacteriophages

## Abstract

Carbapenem-resistant Klebsiella pneumoniae (CRKP) is a major challenge for clinical management worldwide with limited antimicrobial options. Phages are considered an alternative option. Here, we isolated and identified a phage able to lyse ST11-K64 CRKP, the major type in China. This phage has a narrow host range, only lysing ST11-K64 CRKP, and inhibits the growth of host strains for 3 h forming large clear plaques (3.0 to 6.0 mm in diameter) with a surrounding halo. This phage exhibited excellent stability in different temperatures and pH and did not contain any virulence, lysogenic, antimicrobial resistance genes nor tRNA, meeting the criteria for phage therapy. Genomic analysis revealed that it represents a novel species of the *Przondovirus* genus according to ICTV standards. However, phage-resistant bacterial mutants emerged after 4-h exposure. Compared to the parental strain, phage-resistant mutants showed nonmucoid appearance and exhibited significantly reduced virulence for Galleria mellonella larva. Three randomly selected phage-resistant mutants were genome sequenced. Interruption of capsular polysaccharide biosynthesis-associated gene *wcaJ* or *wbaZ* by IS*903B* alone or an IS*903*-formed composite transposon was identified. Interruption of *wcaJ* is a known phage resistant mechanism, while that of *wbaZ* is not. By complementing the intact *wbaZ*, the phage susceptibility was restored, confirming the role of *wbaZ* interruption in phage resistance. This highlights that alteration in the capsular polysaccharide biosynthesis gene cluster, which could be due to transposable elements, is a major mechanism for resistance to *Przondovirus* phages in CRKP. Noncapsule-targeting phages may be combined for improving phage therapy against CRKP.

**IMPORTANCE** Phage therapy is an alternative approach against multidrug resistant microorganisms such as carbapenem-resistant Klebsiella pneumoniae (CRKP), which represents a major challenge for treatment due to very limited options of antimicrobial agents. For optimizing phage therapy, more new lytic phages are needed. Here, we isolated and characterized a phage of a novel species able to rapidly lyse a major type of CRKP without carrying any virulence, lysogenic, antimicrobial resistance genes. This phage is therefore suitable for clinical treatment. However, phage-resistant mutants of CRKP strains were observed after exposure. We found a new mechanism, i.e., interruption of a capsular polysaccharide biosynthesis gene *wbaZ* by an insertion sequence-formed composite transposon. Our study demonstrates the capsular polysaccharide biosynthesis gene cluster as a major source of resistance to certain lytic phages in CRKP. This requires more studies to counter phage resistance. Our studies also highlight the critical role of insertion sequences in phage resistance.

## OBSERVATION

Carbapenem-resistant Klebsiella pneumoniae (CRKP) has been labeled as a “Critical” priority pathogen that poses a serious threat to human health by the World Health Organization on the priority list (1). In addition to the development of new antimicrobial agents, phage therapy plays an increasingly important role in treating infections caused by antimicrobial resistant bacteria, including CRKP (2). Successful cases using phages to treat CRKP infections have been increasingly reported worldwide (3, 4), and two clinical trials of phage therapy against CRKP infections are forthcoming (see www.clinicaltrials.gov/). Along with the increasing interest of phages for clinical use, the number of genome sequences of phages in GenBank has increased exponentially. However, very few phages have been reported to meet the requirements of clinical treatment (5) and have been described in detail for their biological characteristics (6). In addition, phage-resistant bacterial mutants are prone to emerge and such resistance has become a major obstacle to the broad application of phages (7–9). Transposable elements such as insertion sequences and transposons are ubiquitous in prokaryotic genomes and could provide the host bacterial strains genetic plasticity to counter various challenges (10). Here, we report a newly recovered phage that is able to lyse K. pneumoniae of sequence type (ST) 11 and capsular type K64, the dominant CRKP type in China, and meet the biological criteria of phage therapy. We also describe a gene involving in phage resistance in CRKP, which has not been reported before.

We used a ST11 K64 CRKP clinical strain, 135077, which was isolated from urine of a hospitalized patient in 2006, as the host strain for isolating phages. CRKP 135077 was resistant to meropenem (MIC, 4 mg/L). The draft genome sequence of 135077 was obtained using the HiSeq-10X platform (Illumina; San Diego, CA, USA) with a paired-end layout of 150 bp followed by assembly using SPAdes v3.15.3 (11). 135077 belonged to ST11 and K64 type but did not contain a known carbapenemase gene. Instead, this strain had mutations of its OmpK35 porin (Fig. S1), which could result in reduced permeability of carbapenems (12) and carried *bla*_CTX-M-65_ gene encoding an extended-spectrum β-lactamase (ESBL). It has been well reported that the combination of production of ESBLs and porin alterations is able to lead to carbapenem resistance in K. pneumoniae (13–15).

10.1128/msphere.00518-22.3 FIG S1 The frameshift mutation of ompK35 in strain 135077. The ompK35 gene of strain 135077 was identified using Kleborate v2.0.0 (https://github.com/katholt/Kleborate). The complete ompK35 of the reference WP_151502934.1 contains AA at position 85 and 86. In contrast, 135077 contains only one A at the positions with missing another A (shown by *). The frameshift mutation due to the deletion results in translating new amino acids of OmpK35 afterward. Download FIG S1, PDF file, 0.4 MB. Copyright © 2022 Yin et al. 2022 Yin et al. https://creativecommons.org/licenses/by/4.0/ This content is distributed under the terms of the Creative Commons Attribution 4.0 International license.

**FIG 1:**
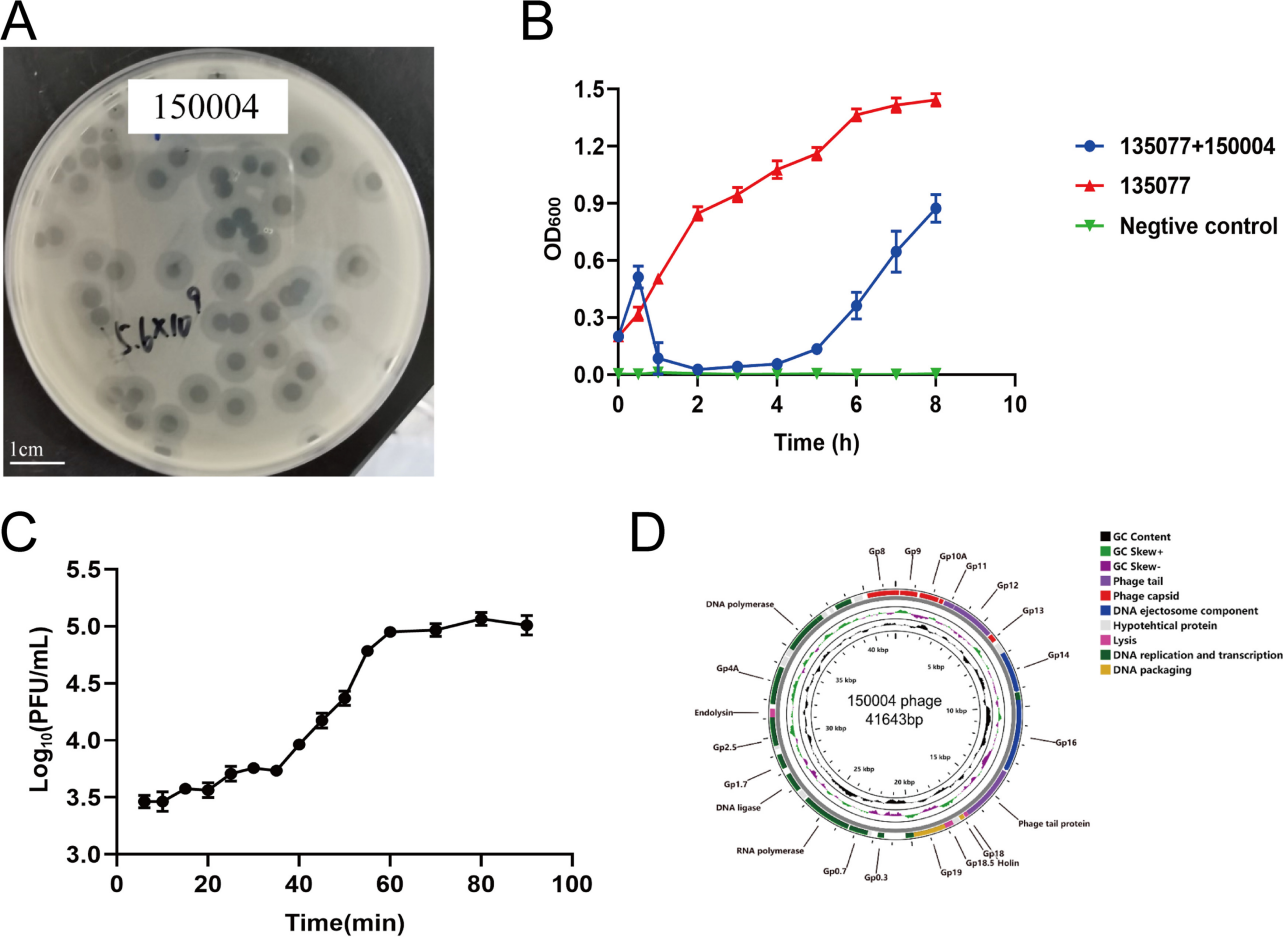
Biological characteristics of phage 150004. Values represent the mean ± standard deviations (*n* = 3). (A) Plaque morphology of phage 150004. Scale bar, 1 cm. (B) The bacteriolytic activity of phage 150004 against strain 135077 at MOI of 0.01 *in vitro*. Negative control is LB broth. (C) One-step growth curve of phage 150004. (D) Annotated gene map of phage 150004. Blocks in various colors represent predicted CDSs encoding products of different functions.

We collected water from a pond in Chengdu, China, in December 2020. We therefore isolated a phage, named 150004 here, from the water sample using methods as described previously (16). This phage is able to lyse strain 135077 with forming bull’s eye-shaped clear plaques (3.0 to 6.0 mm in diameter) that were surrounded by a large opaque halo zone (2.0 to 3.0 mm in diameter) (Fig. 1A). We determined the relative host range of this phage using a limited set of 19 CRKP clinical strains belonging to six STs and 10 capsular types, including five capsular types of ST11 (Table S1). Phage 150004 was able to lyse all ST11 K64 strains but could not lyse strains of any other sequence or capsular types. This suggests that phage 150004 was likely to be specific to ST11 K64 CRKP.

10.1128/msphere.00518-22.1 TABLE S1 The host range of phage 150004. Download Table S1, DOCX file, 0.02 MB. Copyright © 2022 Yin et al. 2022 Yin et al. https://creativecommons.org/licenses/by/4.0/ This content is distributed under the terms of the Creative Commons Attribution 4.0 International license.

We next characterized phage 150004 for its major biological features to demonstrate its potential for clinical applications using a set of experiments performed as described previously (16, 17). In the phage adsorption assay, more than 92% of phage 150004 were rapidly adsorbed to the host strain 135077 in 3 min (Fig. S2). Phage 150004 could grow at temperatures between 0 to 50°C (Fig. S2) and at pH between 5 and 11 (Fig. S2). When the multiplicity of infection (MOI) was 0.01, the titers of phage 150004 reached maximum values after propagation with a titer of approximately 6.22 ± 2.59 × 10^10^ PFU/mL. To assess lytic activity, phage 150004 was added to the log-phase culture of CRKP strain 135077 at MOI 0.01. Strain 135077 was completely lysed by the phage within 1 h and the lysis lasted for approximately 3 h before regrowth (Fig. 1B). The latent period of phage 150004 was 40 min and the burst size was 58 ± 7 progeny phages per infected bacterial cell (Fig. 1C). In the transmission electron microscopy (TEM; Hitachi, Tokyo, Japan) images, phage 150004 exhibited about 10 nm short tails attached to an about 60 nm icosahedral head (Fig. S3). This appearance is consistent with those of phages belonging to the family *Autographiviridae*.

10.1128/msphere.00518-22.4 FIG S2 Characteristics of phage 150004. (A) Absorption rate of phage 150004. (B) Thermal stability of phage 150004. (C) pH stability of phage 150004. Download FIG S2, TIF file, 2.9 MB. Copyright © 2022 Yin et al. 2022 Yin et al. https://creativecommons.org/licenses/by/4.0/ This content is distributed under the terms of the Creative Commons Attribution 4.0 International license.

10.1128/msphere.00518-22.5 FIG S3 TEM morphology of phage 150004. Scale bar, 100 nm. Download FIG S3, TIF file, 2.0 MB. Copyright © 2022 Yin et al. 2022 Yin et al. https://creativecommons.org/licenses/by/4.0/ This content is distributed under the terms of the Creative Commons Attribution 4.0 International license.

We further performed genome sequencing for this phage to investigate its taxonomy and to identify whether it carried genes making it not suitable for phage therapy. Phage DNA was extracted using a phage DNA isolation kit (Norgen Biotek; Thorold, Canada) and was sequenced with HiSeq X10 (Illumina; San Diego, CA, USA). Phage 150004 has a linear double-stranded genome of 41,643 bp with a 52.7% GC content. There are 54 predicted CDSs encoding proteins involved in DNA replication (helicase, endonuclease, exonuclease, DNA polymerase, and RNA polymerase), DNA packaging (terminase large/small subunit), structural proteins (capsid and tail fiber), and host lysis (holin, endolysin, and Rz protein) as annotated by Prokka (18) and Rapid Annotations Subsystems Technology (RAST, http://rast.nmpdr.org/). The gene map of 150004 was constructed using Proksee (https://proksee.ca/) and is shown in Fig. 1D. By BLAST, phage 150004 was closest to phage kpssk3 (GenBank accession no. NC_048114.1) of the genus *Przondovirus* within the family *Autographiviridae* with 95.82% identity and 87% coverage. Phylogenetic tree of 150004 and phages of the genus *Przondovirus* was generated using the one‐click mode of Phylogeny (www.phylogeny.fr) containing a sequence alignment using the MUSCLE and Gblocks programs (19). This phylogenetic analysis revealed phage 150004 belongs to the genus *Przondovirus* (Fig. S4). Phage 150004 had the highest, 91.04%, overall DNA sequence similarity (identity × coverage) with Klebsiella phage 066056 (GenBank accession no MW042808.1), lower than the 95% cutoff for species demarcation defined by the International Committee on Taxonomy of Viruses (ICTV) (20). This suggests that phage 150004 represents a novel species of the genus *Przondovirus* within the family *Autographiviridae*. In addition, phage 150004 had no genes of antimicrobial resistance, virulence factors, lysogen or tRNA.

10.1128/msphere.00518-22.6 FIG S4 Phylogenetic tree of phage 150004 and those of the *Przondovirus* genus. The tree was inferred using the one‐click mode of Phylogeny (www.phylogeny.fr). Phage 150004 is highlighted in blue. Download FIG S4, PDF file, 0.2 MB. Copyright © 2022 Yin et al. 2022 Yin et al. https://creativecommons.org/licenses/by/4.0/ This content is distributed under the terms of the Creative Commons Attribution 4.0 International license.

**FIG 2:**
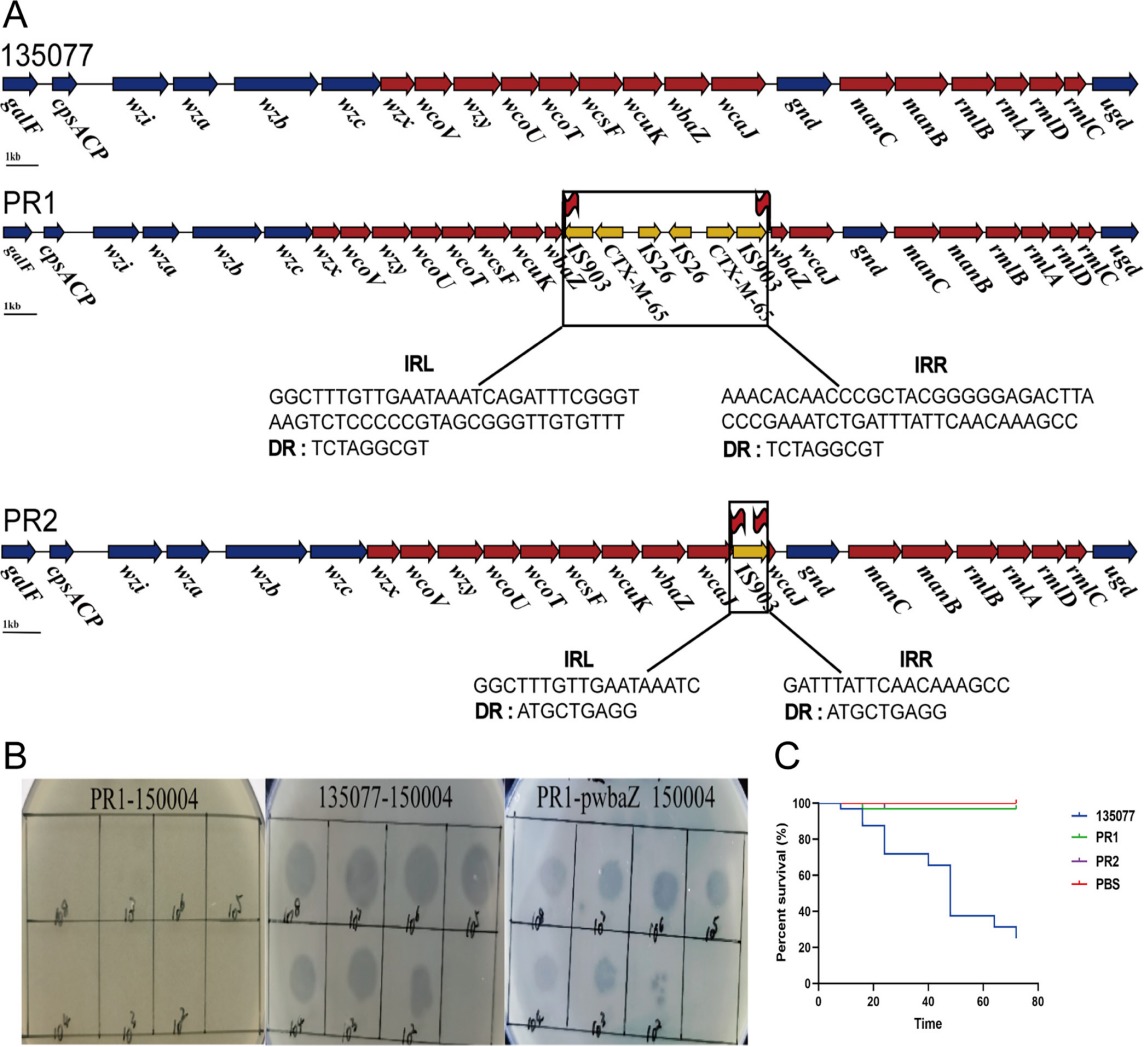
Interruption of the capsular polysaccharide (CPS) gene cluster and its impact on the susceptibility to phage and virulence. (A) The CPS gene cluster in parental strain 135077 and phage-resistant mutants PR1 and PR2. In the CPS gene cluster, conserved genes, variable regions, and insertion sequences are shown in blue, red, and yellow, respectively. The CPS gene cluster contains *galF* (encoding a UDP-glucose pyrophosphorylase), *cpsACP* (encoding an acid phosphatase), *wzi* (related to capsule surface assembly), *wza* (encoding a capsule polysaccharide export protein), *wzb* (encoding a tyrosine phosphatase), *wzc* (encoding a tyrosine-protein kinase), *wzx* (encoding a flippase), *wcoV* (encoding a polysaccharide pyruvyl transferase), *wzy* (encoding an O-antigen and lipid-linked capsular repeat unit polymerase), *wcoU* (encoding a UDP-Glc:α-d-GlcNAc-diphosphoundecaprenol β-1,3-glucosyltransferase), *wcoT* (encoding a glycosyltransferase), *wcsF* (also called *mshA*, encoding a glycosyltransferase), *wcuK* (encoding a glycosyl hydrolase), *wbaZ* (encoding a glycosyltransferase), *wcaJ* (encoding a UDP-phosphate glucose phosphotransferase), *gnd* (encoding a gluconate-6-phosphate dehydrogenase), *manC* (encoding a mannose-1-phosphate guanylyltransferase), *manB* (encoding a phosphomannomutase/phosphoglucomutase), *rmlB* (encoding a dTDP-d-glucose-4,6-dehydratase), *rmlA* (encoding a glucose-1-phosphate thymidylyltransferase), *rmlD* (encoding a dTDP-6-deoxy-l-mannose dehydrogenase), *rmlC* (encoding a UDP-glucose 6-dehydrogenase), and *ugd* (encoding a UDP-glucose 6-dehydrogenase). The functions of CDSs were determined using BLASTp (https://blast.ncbi.nlm.nih.gov/Blast.cgi). *wbaZ* was interrupted by a composite transposon formed by IS*903B* at the 528th nucleotide position in PR1. *wcaJ* was interrupted by IS*903B* of the IS5 family at the 1206th nucleotide position with the 9-bp direct target repeats (ATGCTGAGG) in PR2. Direct target repeats (DR) are shown by flags. The left and right inverted repeats (IRL and IRR) of IS*903B* are also shown. (B) Efficiency of plating (EOP) for phage 150004 against PR1, host strain 135077 and the transformant of PR1 containing pwbaZ. pwbaZ carries an intact of *wbaZ* from strain 135077. (C) Survival curves of Galleria mellonella larva infected with the parental strain 135077 and phage-resistant mutants PR1 and PR2.

As mentioned above, phage 150004 lysed its host strain within 1 h but regrowth of the bacteria strain was observed after 4 h, indicating the presence of phage-resistant mutants. We therefore investigated the mechanisms for resistance to phage 150004. Three phage-resistant mutants, assigned PR1, PR2, and PR3 here, were randomly picked from the LB agar plate containing the mixture of phage 150004 and strain 135077 after overnight incubation at 37°C. The three mutants were further purified by streaking onto LB agar plates without the phage for consecutive three times and their resistance to phage 150004 was further confirmed by spot testing (16). The three mutants were genome sequenced as described above. SNP calling was performed using Snippy v4.6.0 (https://github.com/tseemann/snippy). Comparing to the parental strain, there were no SNPs in PR1, while four SNPs were found in PR2 and PR3, none of which were present in coding sequences. Gene presence/absence was determined using Roary v3.11.2 (21). In PR2 and PR3 the glycosyltransferase-encoding *wcaJ* gene, which involves in capsular polysaccharide (CPS) biosynthesis, was interrupted by insertion sequence IS*903B*. By PCR with self-designed primers wcaJ-IF (CTGTCGTTCCTCTTTTCG) and wcaJ-IR (CCGTTTCACCTCTCCATC) and subsequent Sanger sequencing, the interruption was found to be due to a single copy of IS*903B* (Fig. 2). We and others have previously reported that interruption of the *wcaJ* gene can mediate phage resistance (16, 22, 23). This could explain the phage resistance in PR2 and PR3.

In PR1, *wcaJ* remained intact but another CPS-associated glycosyltransferase gene *wbaZ*, which is located downstream of *wcaJ*, was also interrupted by IS*903B*. However, PCR failed to link the two disrupted fragments of *wbaZ*. We therefore sequenced PR1 using long-read MinION Sequencer (Nanopore; Oxford, UK) and obtained its complete genome by hybrid assembly of short and long reads with Unicycler v0.4.8 (24). We also obtained the complete genome sequence of the parental strain 135077 for comparison. We then identified that *wbaZ* was actually interrupted by a 6,653-bp composite transposon formed by two copies of IS*903B*. This composite transposon also contained two copies of IS*26* and two copies of *bla*_CTX-M-65_ (Fig. 2) and was carried by an 149,214-bp plasmid containing two replicons (IncFII and IncR) in the parental strain 135077 but had mobilized to interrupt *wbaZ* in the chromosome of PR1 from this plasmid using a copy-and-paste model (Table S2). The presence of the composite transposon was verified by two overlapped PCR with self-designed primers binding the two interreupted parts of *wbaZ* and the region between the two copies IS*26* (Fig. S5), respectively, and subsequent Sanger sequencing. As interruption of *wbaZ* has not been reported as a phage resistance mechanism, we performed complementing experiments to verify the role of wbaZ in resistance to phage 150004. We obtained the complete *wbaZ* gene sequence from of the parental strain 135077 by PCR using self-designed primers wbaZ-F-BamHI (CGGGATCCGGTTTGGTATTTATAAG) and wbaZ-R-SacI (AACGAGCTCTCTTTGAACCATTGATA; the restriction sites are underlined). We then cloned PCR amplicons into vector pBC SK (Stratagene; La Jolla, CA, USA), which were transformed into PR1. We obtained a transformant from a LB agar plate containing 40 mg/L chloramphenicol, on which PR1 could not grow. We confirmed the presence of the intact *wbaZ* gene in the transformant by PCR and subsequent Sanger sequencing. We found that the recombined transformant, named PR1::pwbaZ, fully restored its susceptibility to phage 150004 using the spot assay (Fig. 2B). We performed CPS quantification assay in triplicate as described previously (16) and used one-way ANOVA for statistical analysis. We found CPS production of phage-resistant mutant PR1 significantly decreased (mean ± standard deviation [SD], 24.44 ± 1.23 versus 40.47 ± 0.65 mg/L; *P = *0.005) compared with that of the parental strain 135077. In contrast, CPS production of PR1::pwbaZ (mean ± SD, 37.55 ± 0.73 mg/L) was restored to the level comparable to that of 135077. The above findings confirmed that the interruption of *wbaZ* could medicate resistance to phage 150004. In addition to interruption of *wbaZ* and *wcaJ*, alterations of several other genes within the capsule gene cluster, including *mshA* (16), *wcaI* (25), *wbaP* (25–27), *wza* (28), and *wzc* (25, 26, 29) have also been reported to be able to confer resistance to phages in K. pneumoniae. These findings together with previous reports (8, 30, 31) underscore that alterations in capsule genes, especially glycosyltransferases, are important mechanisms to confer phage resistance in bacterial species (7, 32). Transposable elements, in particular insertion sequences, appear to be play a vital role to alter capsule genes by interruption or truncation (16, 33), highlighting that the plasticity offered by these elements is an important mechanism for bacteria to enhance the chance of survival in response to the selection pressure imposed by phages (7).

10.1128/msphere.00518-22.2 TABLE S2 The complete genome and antimicrobial resistance genes of strains 135077 and PR1. Download Table S2, DOCX file, 0.02 MB. Copyright © 2022 Yin et al. 2022 Yin et al. https://creativecommons.org/licenses/by/4.0/ This content is distributed under the terms of the Creative Commons Attribution 4.0 International license.

10.1128/msphere.00518-22.7 FIG S5 Self-designed primers for the two overlapped PCR. The locations of the primers are shown. The two PCR are using 1-IF (TGGAATTGTTGCAGATTGGC)/1-IR (CCTGAAGCTGGGCAAAGTA) and 2-IF (GTCCGGATCATTTCGTCC)/2-IR (CACTGCCATCACCTATAACAATC), respectively. *wbaZ* was interrupted by the IS*903*-formed composite transposon. Download FIG S5, PDF file, 0.07 MB. Copyright © 2022 Yin et al. 2022 Yin et al. https://creativecommons.org/licenses/by/4.0/ This content is distributed under the terms of the Creative Commons Attribution 4.0 International license.

There were two extra copies of *bla*_CTX-M-65_ in PR1, we therefore determined MICs of ceftazidime, cefotaxime and piperacillin-tazobactam using the broth microdilution method according to the Clinical and Laboratory Standards Institute (CLSI) guidelines (34). However, MICs of the tested agents appeared to be the same, ceftazidime (128 mg/L), cefotaxime (>256 mg/L), piperacillin-tazobactam (>256/4 mg/L), for the two strains, suggesting that the two extra copies of *bla*_CTX-M-65_ did not significantly increase the level of resistance to these agents. As CPS is a key virulence factor in K. pneumoniae (35), we also tested PR1 and PR2 (also representing PR3 with the same phage resistance mechanism) for growth, serum resistance, and virulence as described previously (16, 36). Unlike the mucoid appearance of the parental strain 135077, both PR1 and PR2 exhibited the nonmucoid appearance. The virulence experiments were performed using Galleria mellonella larva. For each group, 16 larvae were injected with 10 μL suspension containing 10^8^ CFU/mL of bacterial strain (PR1, PR2, or 135077) or 10 μL PBS as a negative control and the virulence experiments were performed in duplicate, which were pooled (i.e., 32 larvae per group) for statistical analysis using Log-rank test. As expected, the survival rate of larvae (96.88%) infected by PR1 or PR2 was significantly higher than that (25%, *P* < 0.001) by parental strain 135077 (Fig. 2C). However, we did not find differences between the two phage resistant mutants and the parental strain 135077 in growth nor in serum resistance, which performed in triplicate (Fig. S6).

10.1128/msphere.00518-22.8 FIG S6 Some biological differences between the parental strain and phage-resistant mutants. (A) Growth curve of parental strain 135077 and phage-resistant mutants PR1 and PR2. (B) Bacterial serum resistance assay of parental strain 135077 and phage-resistant mutants PR1 and PR2. Download FIG S6, TIF file, 1.9 MB. Copyright © 2022 Yin et al. 2022 Yin et al. https://creativecommons.org/licenses/by/4.0/ This content is distributed under the terms of the Creative Commons Attribution 4.0 International license.

In conclusion, we isolated and characterized a lytic phage against ST11 K64 CRKP, the major type of CRKP in China, meeting the biological criteria of phage therapy. This adds new options to treat CRKP infections. We also found that resistance to phages of the genus *Przondovirus* could be due to interruption of various genes (*wbaZ* and *wcaJ* here as well as *mshA*, *wcaI*, *wbaP*, *wza*, and *wzc* in literature) rather than a specific gene in the CPS biosynthesis cluster. This identifies a target, the CPS biosynthesis gene cluster, for further studies to overcome phage resistance and therefore to improve the potential effect of phage therapy. Alternative, phages targeting the capsule may be combined with those acting on targets for improving phage therapy against multidrug-resistant organisms such as CRKP.

The complete sequence of phage 150004, the complete genome of 135077, the complete genome of PR1, and the draft genome sequences of PR2 and PR3 have been deposited in GenBank under accession numbers OP045496, CP073290-CP073296, CP101726-CP101730, JANHBX000000000, and JANHBY000000000, respectively.
