# Supplementary material for: Interruption of Capsular Polysaccharide Biosynthesis Gene wbaZ by Insertion Sequence IS903B Mediates Resistance to a Lytic Phage against ST11 K64 Carbapenem-Resistant Klebsiella pneumoniae
Source: mSphere. 2022 Nov 15;7(6):e00518-22. doi: 10.1128/msphere.00518-22 (PMC9769513; doi:10.1128/msphere.00518-22)
Supplement: FIG S1 [file msphere.00518-22-s0003.pdf]

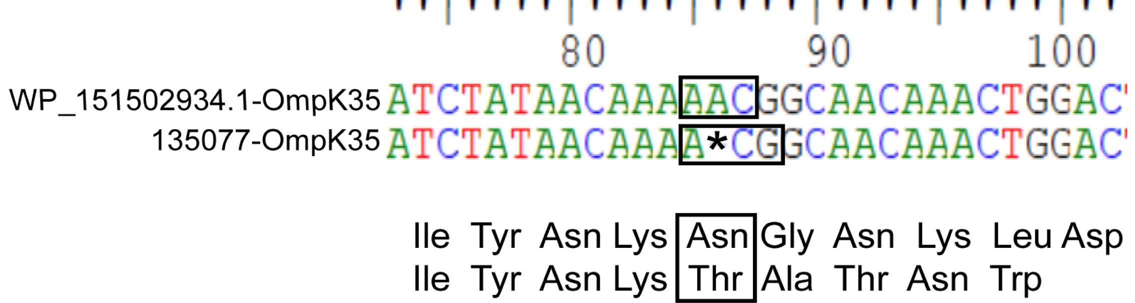
80
90
100
WP_151502934.1-OmpK35 ATCTATAACAAAAACGGCAACAAACTGGAC
135077-OmpK35 ATCTATAACAAAA*CGGCAACAAACTGGAC
Ile Tyr Asn Lys Asn Gly Asn Lys Leu Asp
Ile Tyr Asn Lys Thr Ala Thr Asn Trp
