# Supplementary material for: Interruption of Capsular Polysaccharide Biosynthesis Gene wbaZ by Insertion Sequence IS903B Mediates Resistance to a Lytic Phage against ST11 K64 Carbapenem-Resistant Klebsiella pneumoniae
Source: mSphere. 2022 Nov 15;7(6):e00518-22. doi: 10.1128/msphere.00518-22 (PMC9769513; doi:10.1128/msphere.00518-22)
Supplement: TABLE S1 [file msphere.00518-22-s0001.docx]

Table S1. The host range of phage 150004

| Strain | ST | Capsule type | Carbapenemase genes | Source | Genome accession no. | Susceptibility to 150004 |
| --- | --- | --- | --- | --- | --- | --- |
| 135040 | 11 | KL47 | *bla*_KPC-2_ | sputum | JANHBU000000000 | - |
| 135042 | 11 | KL47 | *bla*_KPC-2_ | sputum | JANHBT000000000 | - |
| 135079 | 11 | KL47 | *bla*_KPC-2_ | secretion | JANHBQ000000000 | - |
| 135044 | 11 | KL64 | *bla*_KPC-2_ | urine | JANHBS000000000 | + |
| 135055 | 11 | KL64 | *bla*_KPC-2_ | sputum | JANHBR000000000 | + |
| 135077 | 11 | KL64 | *-* | urine | CP073290 | + |
| 135025 | 11 | KL64 | *bla*_KPC-2_ | secretion | JANHBV000000000 | + |
| 135080 | 11 | KL64 | *bla*_KPC-2_ | urine | JANHBP000000000 | + |
| 130018 | 11 | KL64 | *bla*_KPC-2_ | blood | JANHBW000000000 | + |
| 140077 | 11 | KL64 | *bla*_KPC-2_ | blood | JANHBO000000000 | + |
| 115057 | 11 | KL39 | *bla*_OXA-181_ | sputum | JANHBK000000000 | - |
| 140127 | 11 | KL21 | *bla*_KPC-2_ | blood | JANHBN000000000 | - |
| 140191 | 11 | KL25 | *bla*_KPC-2_ | blood | JANHBM000000000 | - |
| 020035 | 45 | KL62 | *bla*_KPC-2_ | secretion | CP045988 | - |
| 020116 | 307 | KL102 | *bla*_NDM-1_ | blood | PWCX00000000 | - |
| 090107 | 16 | KL51 | *bla*_NDM-5_ | blood | VCBI00000000 | - |
| 090566 | 789 | KL18 | *bla*_NDM-5_ | blood | JACWGL000000000 | - |
| 140529 | 709 | KL9 | *bla*_KPC-2_ | secretion | JANHBL000000000 | - |
