# Supplementary figures and images for: Interruption of Capsular Polysaccharide Biosynthesis Gene wbaZ by Insertion Sequence IS903B Mediates Resistance to a Lytic Phage against ST11 K64 Carbapenem-Resistant Klebsiella pneumoniae
Source: mSphere. 2022 Nov 15;7(6):e00518-22. doi: 10.1128/msphere.00518-22 (PMC9769513; doi:10.1128/msphere.00518-22)

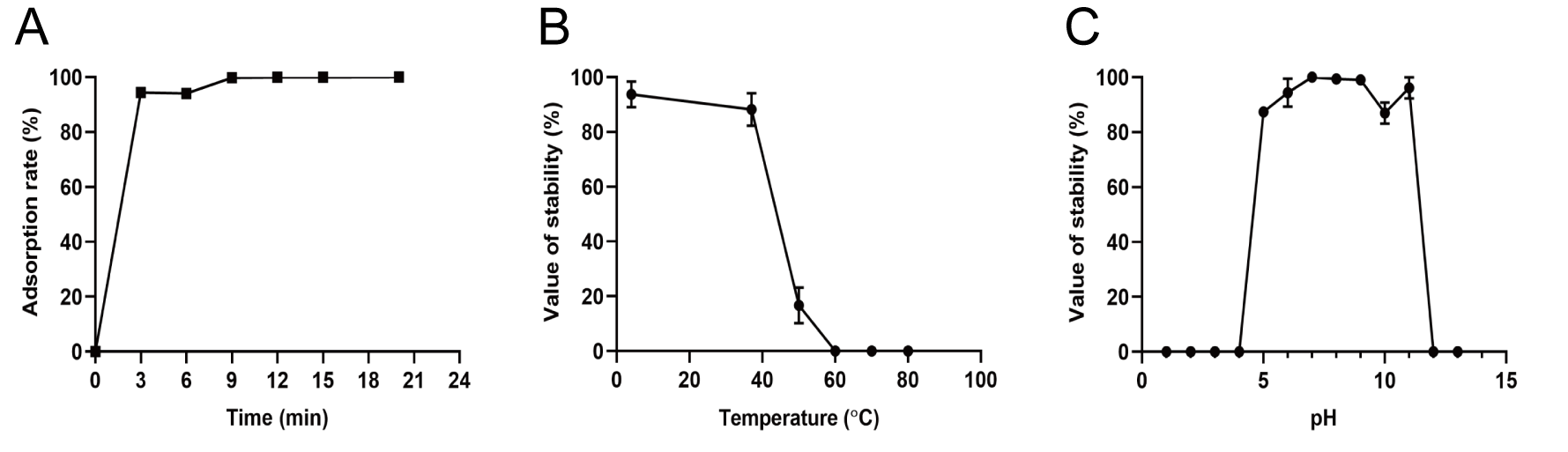

Supplement: FIG S2 [file msphere.00518-22-s0004.tif]

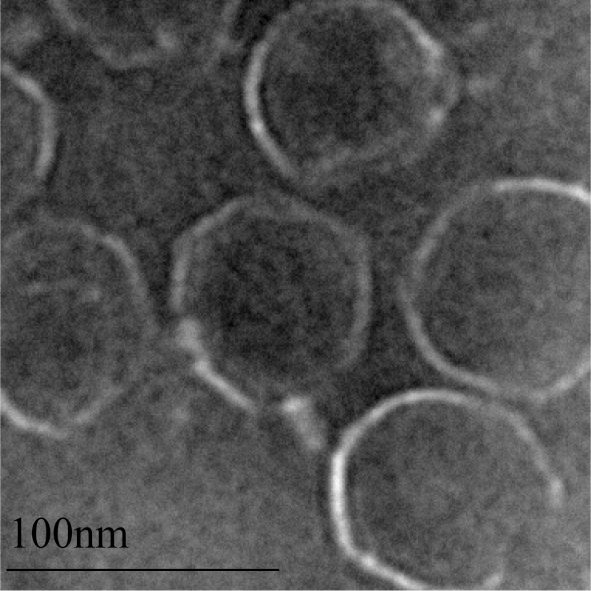

Supplement: FIG S3 [file msphere.00518-22-s0005.tif]

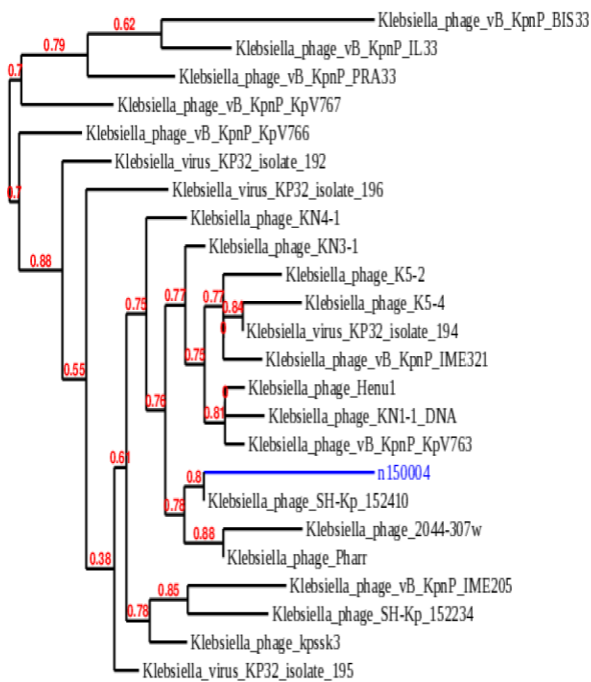

0.01

Supplement: FIG S4 [file msphere.00518-22-s0006.pdf]

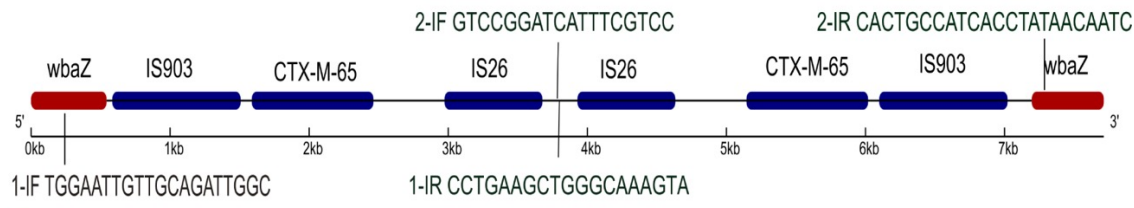

Supplement: FIG S5 [file msphere.00518-22-s0007.pdf]

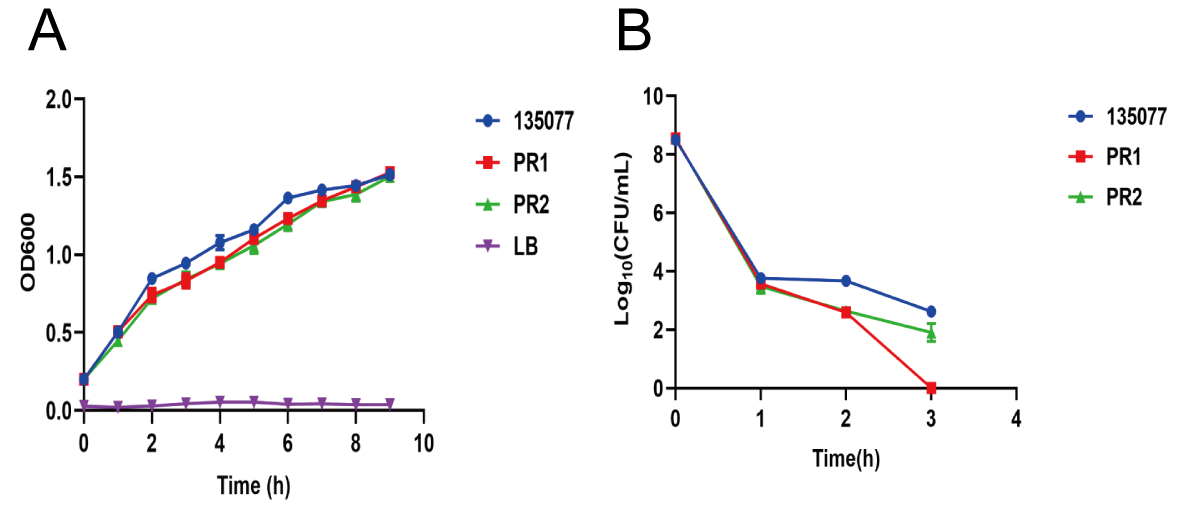

Supplement: FIG S6 [file msphere.00518-22-s0008.tif]
