# Supplementary material for: Interruption of Capsular Polysaccharide Biosynthesis Gene wbaZ by Insertion Sequence IS903B Mediates Resistance to a Lytic Phage against ST11 K64 Carbapenem-Resistant Klebsiella pneumoniae
Source: mSphere. 2022 Nov 15;7(6):e00518-22. doi: 10.1128/msphere.00518-22 (PMC9769513; doi:10.1128/msphere.00518-22)
Supplement: TABLE S2 [file msphere.00518-22-s0002.docx]

Table S2. The complete genome and antimicrobial resistance genes of strains 135077 and PR1.

| Strain | Accession no. | Size, bp | Replicon type |  | Genes mediating resistance to | | | | | | | | |
| --- | --- | --- | --- | --- | --- | --- | --- | --- | --- | --- | --- | --- | --- |
|  |  |  |  | β-lactam | | Aminoglycoside | | Streptomycin | Fosfomycin | Tetracycline | Sulfonamide | Tetracycline | Trimethoprim |
| 135077 | | | | | | | | | | | | | |
| Chromosome | CP073290 | 5,451,354 |  | *bla*_SHV-158_ | | *aadA2* | |  | *fosA6* | *tet(34)* | *sul1* | *tmexD1, tmexD3* |  |
| pCTXM65_135077 | CP073291 | 178278 | IncFII_pHN7A8_, IncR | *bla*_CTX-M-65_ | | |  |  |  |  |  |  |  |
| pVir_135077 | CP073292 | 172,176 | IncFIA, IncFIB | *bla*_TEM-1_ | | |  | *aph(3'')-Ib, aph(6)-Id* |  | *tet(A)* | *sul2* |  | *dfrA14* |
| PR1 |  |  |  |  | | |  |  |  |  |  |  |  |
| Chromosome | CP101726 | 5,459,651 |  | *bla*_CTX-M-65_,  *bla*_SHV-158_ | | | *aadA2* |  | *fosA6* | *tet(34)* | *sul1* | *tmexD1, tmexD3* |  |
| pCTXM65_150040X1B1 | CP101727 | 149,214 | IncFII_pHN7A8_, IncR | *bla*_CTX-M-65_ | | |  |  |  |  |  |  |  |
| pVir_150040X1B1 | CP101728 | 172,348 | IncFIA, IncFIB | *bla*_TEM-1_ | | |  | *aph(3'')-Ib, aph(6)-Id* |  | *tet(A)* | *sul2* |  | *dfrA14* |
